# Diet, Microbiota, and Gut Permeability—The Unknown Triad in Rheumatoid Arthritis

**DOI:** 10.3389/fmed.2018.00349

**Published:** 2018-12-14

**Authors:** Catarina Sousa Guerreiro, Ângelo Calado, Joana Sousa, João Eurico Fonseca

**Affiliations:** ^1^Laboratório de Nutrição, Faculdade de Medicina, Universidade de Lisboa, Lisbon, Portugal; ^2^Instituto de Saúde Ambiental, Faculdade de Medicina, Universidade de Lisboa, Lisbon, Portugal; ^3^Instituto de Medicina Molecular, Faculdade de Medicina, Universidade de Lisboa, Lisbon, Portugal; ^4^Instituto de Bioquímica, Faculdade de Medicina, Universidade de Lisboa, Lisbon, Portugal; ^5^Serviço de Reumatologia e Doenças Ósseas Metabólicas, Hospital de Santa Maria, CHLN, Lisbon, Portugal; ^6^Centro Académico de Medicina de Lisboa, Lisbon, Portugal

**Keywords:** diet, Mediterranean Diet, gut microbiota, gut permeability, rheumatoid arthritis

## Abstract

Growing experimental and clinical evidence suggests that a chronic inflammatory response induced by gut dysbiosis can critically contribute to the development of rheumatic diseases, including rheumatoid arthritis (RA). Of interest, an adherence to a Mediterranean diet has been linked to a reduction in mortality and morbidity in patients with inflammatory diseases. Diet and intestinal microbiota are modifying factors that may influence intestinal barrier strength, functional integrity, and permeability regulation. Intestinal microbiota may play a crucial role in RA pathogenesis, but up to now no solid data has clarified a mechanistic relationship between gut microbiota and the development of RA. Nonetheless, microbiota composition in subjects with RA differs from that of controls and this altered microbiome can be partially restored after prescribing disease modifying antirheumatic drugs. High levels of *Prevotella copri* and similar species are correlated with low levels of microbiota previously associated with immune regulating properties. In addition, some nutrients can alter intestinal permeability and thereby influence the immune response without a known impact on the microbiota. However, critical questions remain to be elucidated, such as the way microbiome fluctuates in relation to diet, and how disease activity may be influenced by changes in diet, microbiota or diet-intestinal microbiota equilibrium.

## Diet, Microbiota, and Gut Permeability—The Unknown Triad in Rheumatoid Arthritis

A growing body of experimental and clinical evidence suggests that a chronic inflammatory response induced by gut dysbiosis can critically contribute to the development of a number of rheumatic diseases, including RA (1–3). Of interest to this discussion, some RA patients suffer from clinical or subclinical gut disturbances (4). It has been hypothesized that at some point of the preclinical phase of RA, at the level of the mucosal surface, interactions between microbes, and other potential environmental factors (e.g., diet, physical, and emotional stress) as well as host factors lead to mucosal inflammation and to the breaking of immune tolerance. This mucosal inflammation may enhance local—and then systemic—immune disturbances, through mechanisms that may include molecular mimicry or facilitation of direct autoimmunity to self-antigens (5, 6). Breaching this single layer of epithelium can lead to pathological exposure of the highly immunoreactive subepithelium to foreign antigens in the lumen (4). The equilibrium between tolerance and immunity to non-self-antigens can be ruptured by this increased intestinal permeability, which in many cases may facilitate absorption of antigens and contribute to the persistence and exacerbation of some immune mediated diseases, including RA (7).

Two major altering factors that may influence barrier strength and functional integrity, with an effect in intestinal permeability regulation, are diet and intestinal microbiota. These factors may allow the entry of external antigens from the gut lumen into the host (6, 8, 9). Both are life style related, which suggests that environmental factors might influence the function of the intestinal barrier and thus, influence immune health and the onset and activity of diseases such as RA (9).

### Intestinal Microbiota and Rheumatoid Arthritis

The intestinal tract harbors the largest bacterial community associated with the human body. Everyone carries up to a few hundred species of intestinal bacteria (9). Over 90% of intestinal bacteria belong to the *Bacteroidetes* and *Firmicutes* phyla, but other phyla like *Proteobacteria, Actinobacteria, Fusobacteria, Verrucomicrobia*, and *Cyanobacteria* also play a crucial role in the maintenance and regulation of homeostasis in intestinal microflora (3, 7). Among other functions, the intestinal microbiota is a critical factor for the homeostasis of the host immune system and as such, any alteration on the gut microbiota may impact on the host immune response (10, 11).

Human studies revealed that patients with RA display significant differences of the intestinal microbiota and a decreased gut microbial diversity in comparison to healthy controls (3), both related with disease duration and autoantibody levels (3, 12, 13). Patients with RA, particularly erosive patients, carry a distinctive enterotype of gut microbiota characterized by a lower abundance of bacteria belonging to the family *Bifidobacterium* and *Bacteroides* (12, 14, 15) and, at least at early stages of the disease, an abundance of *Prevotella copri* (14, 16).

The association between an imbalance of the intestinal microbiota and RA has been suggested to happen due to different mechanisms that may impact the host immune system and its function, including:

- the activation of antigen-presenting cells (APCs), such as the dendritic cells, which can impact the cytokine production and antigen presentation. Such disturbances can further modulate the host immune response by, for instance, impinging on T cells differentiation and function. These microbiota-induced effects can be mediated through pathogen recognition receptors, key innate immune receptors, capable of perceiving pathogen-associated molecular patterns, such as the toll-like receptors (TLRs) (10);
- the ability to promote the citrullination of peptides via the enzymatic action of peptidyl-arginine deiminases (PADs). Concerning this, it is of interest that the intestinal epithelium is a major producer of citrulline in the human body (4). Furthermore, PAD is active in the human intestine and the intestinal microbiome may also encode active microbial PADs (4). Thus, the intestine may stand as a source of citrullinated peptides, along with other mucosal surfaces. In this respect, peptide citrullination by the bacterial PAD enzyme expressed by *P. gingivalis* has been suggested to strongly contribute for the close association between periodontitis, an inflammatory disease of the oral mucosa, with an increased susceptibility to RA (10, 17);
- antigenic mimicry, which can result from similarities existing between foreign antigens and self-antigens, that then evoke the activation of pathogen-derived autoreactive T and B cells and thus lead to autoimmunity (10);
- impact on the permeability of the intestinal mucosal, by modulating the expression of tight junction (TJ) proteins;
- and control of the host immune system. This effect can be exerted, for instance, by modulating T cells differentiation and unbalancing the homeostasis between T helper type 17 (Th17) cells and T regulatory (Treg) cells. In RA mice models, it has been shown that specific alterations in the intestinal microbiota may favor the pathophysiological action of Th17 cells in detriment of the suppressive action of Treg cells, consequently, promoting Th17-mediated mucosal inflammation (10).

Overall, the elicited immune responses may be dependent on the presence of certain genera/species. *Bacteroides fragilis*, for example, can stimulate Th1-mediated immune responses in the initial stages of colonization by producing polysaccharide *A. Collinsella sp* and may contribute to RA pathogenesis by increasing gut permeability, lowering the expression of TJ proteins and influencing the epithelial production of IL-17A (10, 12). Butyrate producing microbes, such as *Clostridia, Faecalibacteria*, and some species of *Lachnospiraceae* may also play a crucial role in keeping the integrity of intestinal epithelia, having a documented anti-inflammatory effect in the context of rheumatic diseases, including RA (3, 14).

In addition, the idea that the onset of autoimmunity may be related to gastrointestinal tract is supported not only by the fact that microbiota composition in subjects with RA differs from controls, but also by the observation that altered microbiome can be partially restored after prescribing disease modifying antirheumatic drugs (3).

## Prevotella: Conflicting Data Concerning Rheumatoid Arthritis

Adding an additional level of complexity, it is becoming increasingly clear that the close interplay between variations in microbiota composition and RA may not be so simple. As a paradigmatic example, several conflicting associations have been observed regarding *Prevotella* species and RA pathogenesis. In fact, although some studies in RA patients display an association of *Prevotella* species as a contributive risk factor to the onset of the disease, contrarily, others have suggested that it may be protective (10). The existing data sustain that the gut microbiota of established RA patients exhibits a lower abundance of *Prevotella* species (15). However, Scher et al. (18) reported that individuals with early RA were more likely to harbor *Prevotella copri* (PC) compared to controls. Also, Maeda et al. (19) observed PC in abundance within gut microbiota in Japanese patients with early RA. According to these authors this species has a relevant impact on the inflammatory response, by inducing Th17 related cytokines, such as IL-6 and IL-23, and an increase on the intestinal permeability as well, with influence on bacteria penetration throughout the body. In addition, high levels of PC and similar species are correlated with low levels of protective microbiota, which are believed to regulate the immune system (20). An abundance of PC in early RA stages may very likely stand as an important mechanism that links dysbiosis with arthritis pathogenesis (10).

Marietta et al. (16) also focused their attention at the *Prevotella* species. In an experimental study they showed that *Prevotella histicola* can lead to a lower gut permeability by increasing the expression of enzymes required to produce antimicrobial peptides, as well as, of TJ proteins (zonula occludens 1 and occludin). These authors suggest this species to have immunomodulating properties, by generating Treg cells and increasing the transcription of interleukin-10 (16).

It is worth noticing that more than 40 different *Prevotella* species exist, with a vast and different array of genome repertoires among strains and between *Prevotella* species and hosts. This genetic high diversity found within *Prevotella* species might explain the highly different behavior observed when considering the genus-level identification (18), which possibly explain part of inconsistences observed when relationship between diet and health/disease is tested (10, 21). It's still missing, however, a question that so far any study has placed, which is of whether of the same genus are consistently linked to dietary patterns or equally responsive to diet variations and at a more complex level, in what manner such relationship may influence the disease behavior (22).

## Diet and Rheumatoid Arthritis

Discussion about possible mechanisms by which diet influence RA, its effect on intestinal microbiota and gut permeability are also a current hot topic. Literature has been showing that individual diet content may have an important impact on microbiota and metabolome expression, thereby influencing intestinal integrity and permeability (11, 23, 24).

Clustering of the human gut microbiota, designated enterotypes, was first described in 2011 (25, 26). One of the largest bacterial groups that can be found in the gut are the *Bacteroidetes*. Amongst this phylum, there are a lot of genera which are diet-responsive. Particularly, the increase in *Prevotella* and *Bacteroidetes* can be associated to a high-fiber diet and to the consumption of a diet rich in both fat and animal protein, respectively. (8, 19).

The *Bacteroides*-driven enterotype is reported to be predominant in individuals consuming more animal protein and saturated fats (western diet), becoming clearly that some kind of nutrients/food may enhance this type of enterotype. In contrast, the *Prevotella*-driven enterotype is poorly represented in these individuals but present at high values in individuals consuming carbohydrates, simple sugars and fiber, suggesting an association with a carbohydrate-based diet (8, 27).

The food patterns of the populations who lived around the Mediterranean sea during the 60's, inspire what is, nowadays, commonly known as The Mediterranean Diet (MD), which is, proven, one of the healthiest dietary pattern existing (28–30). The essence of this diet consists in the high consumption of products like fruits, vegetables, legumes, unrefined cereals and nuts, the moderate consumption of fish, poultry and dairy products as cheese and yogurt, and the low consumption of red meat products. Olive oil is used as the main edible-fat source and wine is consumed in a regular, but moderate, basis (29, 30). The reduction in overall mortality and morbidity has been linked to a greater adherence to the MD and this food pattern is highly proposed as a beneficial dietary approach for patients with inflammatory diseases (30, 31).

According to some authors, important RA disease characteristics namely, inflammatory activity disease, physical function and vitality, may be clinically related to MD pattern, particularly when compared to a western diet (10, 32). No association has been proved regarding the susceptibility to disease, nonetheless. On the Skoldstam's study (33), after 12 weeks of intervention, patients in the MD group showed a significant improvement in DAS-28 (Disease Activity Score-−28 joints), HAQ (Health Assessment Questionnaire), whereas subjects in the control group displayed no significant changes in these parameters. In another study, developed by Mckellar et al. (34) and Tedeschi and Costenbader (35), after 6 months, patient global pain VAS (Visual Analog Scale), and morning stiffness were significantly improved in the intervention arm when compared to the control arm (35). It is important to note that RA disease activity was significantly decrease in patients with adequate level of typical MD antioxidant as vitamin C, retinol, and uric acid (32).

An association between MD and short chain fatty acids (SCFAs) production, is well known and theoretically plausible. Rich fiber foods such as fruit, vegetables, and legumes, all regularly consumed by individuals who practice a MD pattern, have the ability to become degraded by *Firmicutes* and *Bacteroidetes* bacteria, leading to high feacal SCFA (30, 36). Fiber is the most well-known nutrient with an important impact on microbiota. Diets rich in this nutrient increase SCFA producing bacteria, with benefits on intestinal barrier structure (11). Particularly the butyrate strengthens the barrier by increasing TJ protein expression and transepithelial electrical resistance (TER), and by consequently decreasing intestinal permeability and bacterial translocation. This has been shown to prevent the activation of effector T cells and to abrogate the manifestation of undesirable local and systemic inflammatory responses (9, 11, 37). The changes in gut microbial ecology and associated SCFA driven immune modulation can, in a logical way, explain the mechanisms behind clinical amelioration of RA in individuals exposed to MD. Despite of that, the validation in humans it's still needed (36).

Overall, additional research is necessary to elucidate the links between the health-promoting food pattern of the MD with gut microbiota characteristics (30).

Four small RCT have also studied the potential benefits of probiotics in RA. Probiotics are living organisms with a well-documented range of health benefits, associated not only with antimicrobial effects, but also to immune system, or with gut permeability (9, 10, 38, 39). In two small RCTs, *Lactobacillus casei* was shown to significantly reduce the expression of pro-inflammatory cytokines, namely IL-6 and TNF-α, and COX-2 activity, alleviating the manifestations of RA (3, 40, 41). Another study (42), with an association of probiotics (*Lactobacillus acidophilus, Lactobacillus casei*, and *Bifidobacterium bifidum*) resulted in improved DAS28 and a lower high sensitivity C-reactive protein concentrations when compared with placebo. Another RCT, testing a different *Lactobacillus* strain (*L. rhamnosus*), did not demonstrate any efficacy after one year of use (32, 43). In addition, several *in vivo* studies have assayed the use of probiotics as a therapeutic intervention. *Lactobacillus casei* was observed to attenuate symptoms in the mouse collagen-induced arthritis model by changing Th17 cells production, via no expression of IL-6, IL-17 and IL-23 cytokines (14, 15, 44). Same results were reported when using the *Lactobacillus* GG strain in the antigen-induced arthritis model in Lewis rats (14, 15, 45). Moreover, the use of *E.coli* Nissle 1917, *Bifidobacterium infantis* and *Lactobacillus plantarum* appears to have a positive impact on barrier strength with benefits on expression and production tight junction proteins (6, 9). By contrast, *Lactobacillus bifidus* showed a probable ability to promote joint swelling in germ free mice, by TLR2/TLR4 activation (3, 34, 46). Although these studies have globally suggested the potential benefits of probiotic supplementation in RA, until now the evidence is not considered strong enough to propose the inclusion of probiotic supplements in the diet of RA patients as part of their disease management strategy (47).

## Specific Nutrients and Gut Permeability

There are also some nutrients that can alter intestinal permeability and thereby influence the immune response without a known impact on the microbiota (Table 1). The depletion of some amino degrade carbohydrates acids like glutamine (55) or tryptophan (11) has been associated with a lower barrier function, translated in increased gut permeability. Vitamin D (7, 9, 11, 18) or polyphenols, as quercetin, myricetin, kaempferol, or curcumin have been shown to participate in the regulation of the intestinal barrier too, once they promote TER enhancement and a higher expression of TJ proteins, likeZO-1 and claudin-1. Also, a depletion of zinc, a micronutrient known to be essential for cell survival and function, has been shown to increase intestinal permeability (59). Interestingly most of these nutrients are commonly abundant in a MD pattern, which as referred before, is known as a potentially beneficial diet in RA. Apart from these nutrients, other food compounds, mostly present at western diets such as fatty acids [specially from high fat diets; (48)], alcohol (11), additives used in food industry (53), gliadin [protein present in wheat and several other cereals; (50, 51)], chitosan (52) or even some food processing methods using different microbial and fungal strains [that promotes eventual horizontal gene exchange; (60)], are known to negatively regulate barrier function via compromising its integrity as they alter TJ proteins expression and distribution, decrease TER and favor the growth of pathogenic or opportunistic bacteria (9, 61).

**Table 1:** Nutrients/Compounds that increase or decrease the intestinal permeability.

**GUT PERMEABILITY**	
**Increase**	**Decrease**
High-fat diets (48) Milk fat (11, 49) Fatty acids: capric acid, lauric acid, long-chain fatty acids, eicosapentaenoic acid, γ-linoleic acid, and docosahexaenoic acid (11, 49) Gliadin (50, 51) Alcohol (and acetaldehyde; 37) Chitosan (52) Additives used in food industry: salt, sugar, emulsifiers and surfactants, organic solvents, and microbial transglutaminase (53) Vegetables extracts (54)	Glutamine (55, 56) Tryptophan (11) Vitamin D (7, 8, 11, 18) Polyphenols: quercetin, myricetin, and kaempferol (57, 58) Curcumin (11, 56) Zinc (59)

However, the real-life impact of all these nutrients on the intestinal permeability and the immune response in RA patients is still undisclosed (62).

## Conclusions

**Figure 1:**
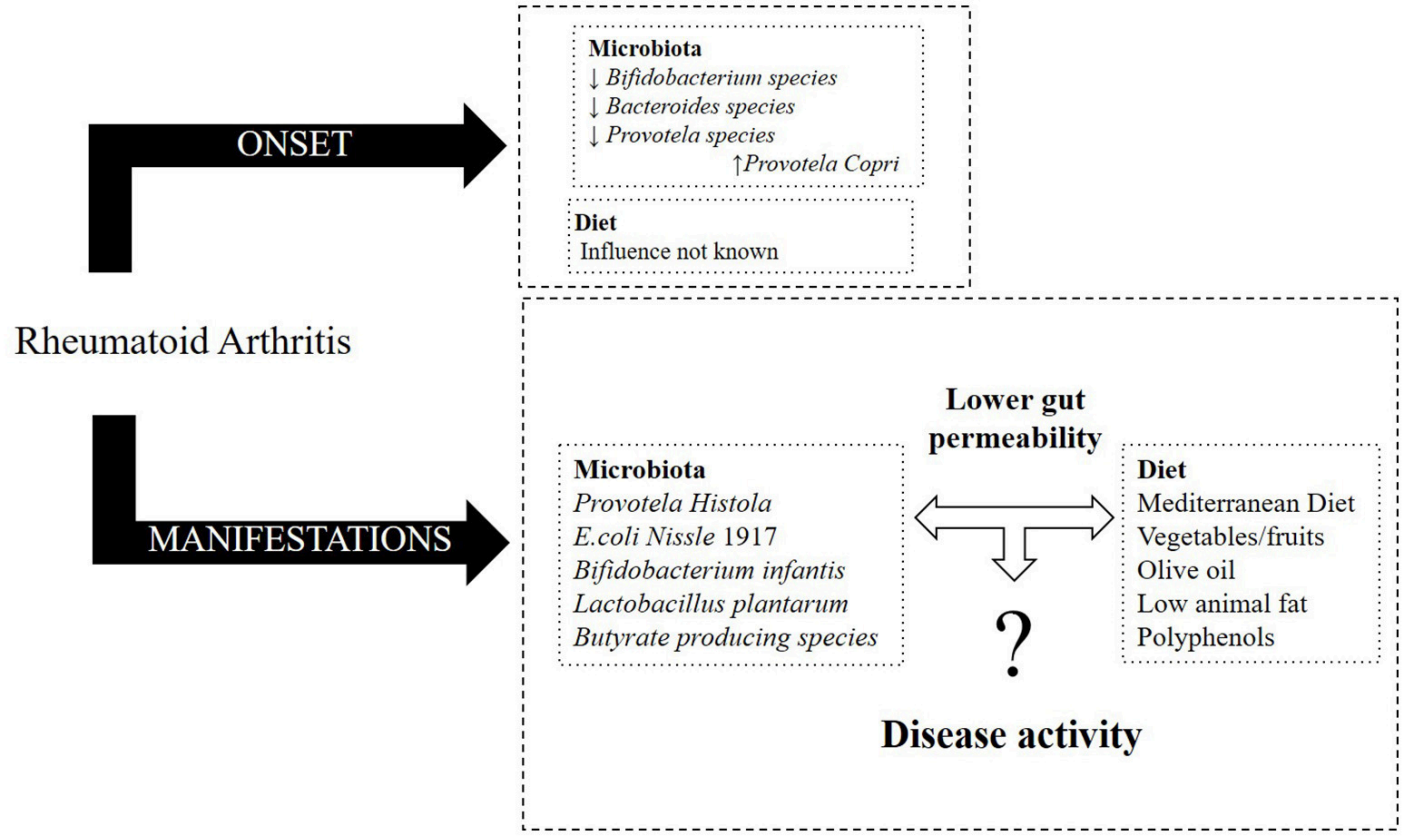
Effect of microbiota and diet in RA (onset and its manifestations).

Overall, it is now known that RA patients display a distinct gut microbiota composition at the moment of diagnosis in comparison to healthy individuals. Moreover, the microbiota composition of RA patients suffers further changes as the disease progresses. On the other hand, diet, especially the MD pattern, seems to influence the physiopathology of RA, potentially lowering disease activity, and improving the outcome (Figure 1).

However, critical questions remain to be elucidated, keeping the research agenda active in this field:

1. Are the clinical benefits observed with MD associated with the selective growth of a “healthy gut microbiota”? This possible association remains to be documented.
2. What is the relevance of intestinal permeability in this equation since both diet and microbiota seem to change its functionality? It is still unclear if intestinal permeability is directly affected independently by both variables or if the mechanism is sequential, depending on the influence of diet on microbioma.
3. How the microbiome fluctuates in relation to diet, and how disease activity may be influenced by changes in diet, microbiota or diet-intestinal microbiota equilibrium.

## Author Contributions

All authors listed have made substantial, direct, and intellectual contribution to the work and approved it for publication. CG, ÂC, JS, and JF specifically in concept. CG design and literature search. CG, ÂC, and JS data processing and writing manuscript. AC and JF critical review and in supervision.

### Conflict of Interest Statement

The authors declare that the research was conducted in the absence of any commercial or financial relationships that could be construed as a potential conflict of interest.
